# Bee chitosan nanoparticles loaded with apitoxin as a novel approach to eradication of common human bacterial, fungal pathogens and treating cancer

**DOI:** 10.3389/fmicb.2024.1345478

**Published:** 2024-03-15

**Authors:** Mohamed Sharaf, Abdullah A. Zahra, Maha Alharbi, Alsayed E. Mekky, Abdelrazeq M. Shehata, Abdulsalam Alkhudhayri, Ahmed M. Ali, Ebtesam A. Al Suhaimi, Shadi A. Zakai, Norah Al Harthi, Chen-Guang Liu

**Affiliations:** ^1^Department of Biochemistry and Molecular Biology, College of Marine Life Sciences, Ocean University of China, Qingdao, China; ^2^Department of Biochemistry, Faculty of Agriculture, AL-Azhar University, Cairo, Egypt; ^3^Department of Plant Protection, Faculty of Agriculture, AL-Azhar University, Cairo, Egypt; ^4^Department of Biology, College of Science, Princess Nourah bint Abdulrahman University, Riyadh, Saudi Arabia; ^5^Department of Botany and Microbiology, Faculty of Science, Al-Azhar University, Cairo, Egypt; ^6^Department of Animal Production, Faculty of Agriculture, Al-Azhar University, Cairo, Egypt; ^7^Department of Biology, College of Sciences, University of Hafr Al Batin, Hafar Al Batin, Saudi Arabia; ^8^Department of Biology, Shaqra University, Shaqra, Saudi Arabia; ^9^Vice Presidency for Scientific Research and Innovation, Imam Abdulrahman Bin Faisal University, Dammam, Saudi Arabia; ^10^King Abdulaziz and his Companions Foundation for Giftedness and Creativity “Mawhiba”, Riyadh, Saudi Arabia; ^11^Department of Clinical Microbiology and Immunology, Faculty of Medicine, King Abdulaziz University, Jeddah, Saudi Arabia; ^12^Department of Clinical Laboratory Sciences, College of Applied Medical Sciences, Taif University, Taif, Saudi Arabia

**Keywords:** antimicrobial resistance, *Apis mellifera*, apitoxin, bee’s chitosan NPs, colon cancer, drug delivery system

## Abstract

Antimicrobial resistance is one of the largest medical challenges because of the rising frequency of opportunistic human microbial infections across the globe. This study aimed to extract chitosan from the exoskeletons of dead bees and load it with bee venom (commercially available as Apitoxin [Api]). Then, the ionotropic gelation method would be used to form nanoparticles that could be a novel drug-delivery system that might eradicate eight common human pathogens (i.e., two fungal and six bacteria strains). It might also be used to treat the human colon cancer cell line (Caco2 ATCC ATP-37) and human liver cancer cell line (HepG2ATCC HB-8065) cancer cell lines. The x-ray diffraction (XRD), Fourier transform infrared (FTIR), and dynamic light scattering (DLS) properties, ζ-potentials, and surface appearances of the nanoparticles were evaluated by transmission electron microscopy (TEM). FTIR and XRD validated that the Api was successfully encapsulated in the chitosan nanoparticles (ChB NPs). According to the TEM, the ChB NPs and the ChB NPs loaded with Apitoxin (Api@ChB NPs) had a spherical shape and uniform size distribution, with non-aggregation, for an average size of approximately 182 and 274 ± 3.8 nm, respectively, and their Zeta potential values were 37.8 ± 1.2 mV and − 10.9 mV, respectively. The Api@ChB NPs had the greatest inhibitory effect against all tested strains compared with the ChB NPs and Api alone. The minimum inhibitory concentrations (MICs) of the Api, ChB NPs, and Api@ChB NPs were evaluated against the offer mentioned colony forming units (CFU/mL), and their lowest MIC values were 30, 25, and 12.5 μg mL^−1^, respectively, against *Enterococcus faecalis*. Identifiable morphological features of apoptosis were observed by 3 T3 Phototox software after Api@ChB NPs had been used to treat the normal Vero ATCC CCL-81, Caco2 ATCC ATP-37, and HepG2 ATCC HB-8065 cancer cell lines for 24 h. The morphological changes were clear in a concentration-dependent manner, and the ability of the cells was 250 to 500 μg mL^−1^. These results revealed that Api@ChB NPs may be a promising natural nanotreatment for common human pathogens.

## GRAPHICAL ABSTRACT

Schematic 1: **(A)** Illustration of the preparation of Api@ChB NPs from honeybee *Apis mellifera* chitosan; and **(B)** Api@ChB NPs bactericidal mechanism of eradication. (Created with BioRender.com).

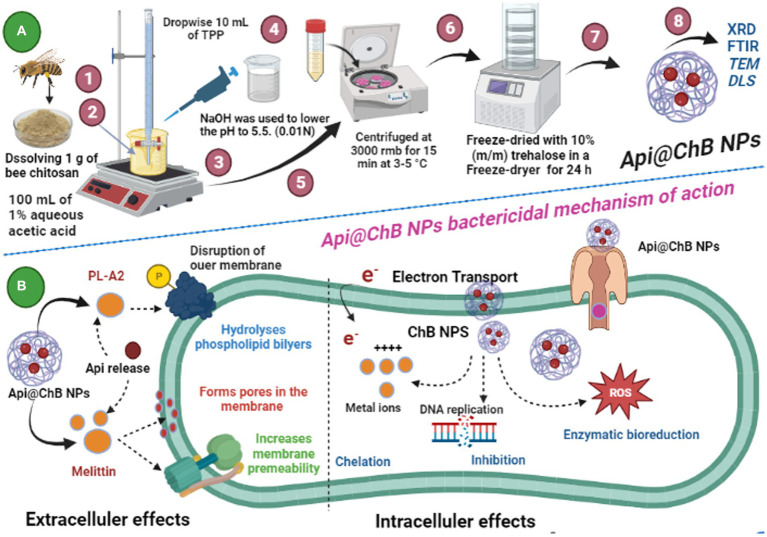

## 1 Introduction

Multidrug-resistant (MDR) organisms bacteria-have developed so much resistance to specific medicines that the medicines can no longer be used to contain or eradicate the bacteria (Breijyeh et al., 2020). Statistics on illnesses caused by MDR bacteria indicate that bacterial resistance to antibiotics has increased exponentially in recent years, and it is anticipated that MDR bacteria will be responsible for 10 million deaths by the year 2050 (Frattari et al., 2019).

The ineffectiveness of antibiotics can be due to low bioavailability, a poor ability to reach infection sites, and the development of MDR bacteria, or all three. Drug-delivery systems may solve this problem (Li et al., 2017). There is a growing need for innovative delivery techniques to improve the therapeutic efficiency of existing antibiotics, particularly those used against MDR bacteria (Canaparo et al., 1991).

Chitosan is a natural polysaccharide that has been deacetylated from chitin (poly-β-(1 → 4)-Nacetyl-D-glucosamine). Chitosan has been employed as a mechanism for delivering medications or in drug formulations in a variety of fields, including agriculture, the cosmeceutical industry, food protection industry, biomedicine, and pharmaceutical industry (Chen et al., 1998; Mezzana, 2008; Morganti, 2009; Francis et al., 2015).

Increasingly, chitin and chitosan are being used as drug-delivery systems and in nanocomposites (Aranaz et al., 2009), perhaps because of the functions enabled by their physicochemical behaviors. In addition, a biopolymer containing chitosan was used as an antibacterial agent (Xu et al., 2021) and also had capabilities as an antifungal agent (Pereda et al., 2011).

The biopolymer chitin is found in a wide variety of organisms, including the exoskeletons of crustaceans (e.g., lobster, shrimp, krill, barnacles, crayfish), mollusks (e.g., octopuses, cuttlefish, clams, oysters, squid, snails), algae (e.g., diatoms, brown algae, green algae), and insects (e.g., houseflies, silkworms, ants, cockroaches) (Joseph et al., 2021; Crognale et al., 2022). Bees use chitin to limit the growth of their exoskeletons (Carpena et al., 2020).

*Apis mellifera* (Apidae family), or honey bees, produce substances that have traditionally been utilized in nutritional additives that promote health (Thakur and Nanda, 2020). These compounds have a variety of biological functions, including antibacterial, anti-inflammatory, anticancer, and antioxidant effects (Nainu et al., 2021; Ranneh et al., 2021). Among the physiologically active components found in bee products are proteins, peptides, minerals, flavonoids, terpenes, fatty acids, and phenolic compounds (Huang et al., 2014).

Bee venom, commercially available as Apitoxin (Api), is a biotoxin generated in honey bees’ venom glands, which are located at the base of their abdomens; it is one of the bees’ most important products (Carpena et al., 2020). Api, a complex combination of proteins, enzymes, and amines, affects the host locally and systemically (Komi et al., 2018). Melittin, apamin, adolapin, and mast cell degranulating peptides are peptides found in Api (El-Seedi et al., 2020), and each has been investigated for the possibility that it might have a therapeutic use.

Api may also be a promising option for the treatment of antimicrobial infections (Otręba et al., 2021). The antibacterial activity of Api has been demonstrated against various strains of Gram-positive bacteria, such as *Staphylococcus aureus* (Han et al., 2016), *Staphylococcus hyicus*, *Staphylococcus chromogenes*, *Streptococcus salivarius*, *Streptococcus sanguinis*, *Streptococcus sobrinus*, *Streptococcus mitis*, *Streptococcus mutans*, *Enterococcus faecalis,* and *Bacillus subtilis* (Kim et al., 2006; Perumal Samy et al., 2007; Leandro et al., 2015). Its activity has also been proven against Gram-negative bacteria, such as *Klebsiella pneumoniae*, *Salmonella typhimurium,* and *Escherichia coli* (Zolfagharian et al., 2016). Gram-positive bacteria have been shown to be more susceptible to Api than Gram-negative bacteria. Furthermore, both Api and melittin have shown varying antifungal activity against different species of pathogenic fungi, such as *Trichophyton mentagrophytes*, *Trichophyton rubrum*, *Candida albicans*, *Candida krusei*, *Candida parapsilosis*, and *Candida tropicalis* (Al-Ani et al., 2018). Since the antibacterial property of bee products is dependent on their chemical composition, further study is required to standardize the chemical makeup of these compounds before they may be utilized to treat bacterial infections (Otręba et al., 2021). The discovery of antimicrobial medications encourages the quest for novel alternatives with distinct action mechanisms. Both chitosan and Api have demonstrated highly promising and potent capabilities as alternatives for treating bacteria and fungi (Ilium, 1998; Elella et al., 2018; Goda et al., 2021).

Furthermore, extensive studies have been conducted on the Api and its constituents, such as melittin, as potential therapeutic agents and inhibitors for various types of tumors. These investigations have revealed multiple molecular mechanisms through which BV and its components exhibit potential anticancer properties and cytotoxic agents with a broad spectrum of activity against different types of tumor cells (Oršolić, 2012; Gajski and Garaj-Vrhovac, 2013; Aufschnaiter et al., 2020). Additionally, the use of chitosan nanoparticles as carriers and enhancers for various animal venoms, including snake (PoMohammadur Dounighi et al., 2015), scorpion (Fouda et al., 2023), and bees (El-Didamony et al., 2022), was shown to be effective in their assessment as potential anticancer agents. In regards this study aimed to extract chitosan nanoparticles from the exoskeletons of dead bees (ChB NPs) for use as a novel drug-delivery system that might be effective against human fungal and bacterial pathogens. This would occur via ionic interactions of charged groups situated on chitosan surface molecules with microbial walls that could promote the hydrolysis of peptidoglycans and the seepage of internal cytoplasmic substances, ultimately leading to microbial death. In order to increase the antimicrobial activity and cell permeability and the formation of holes in the outer membrane of microbes, we loaded Api into ChB NPs.

## 2 Materials and methods

### 2.1 Apitoxin

Apitoxin was collected from the carniolan honeybee hybrid *Apis mellifera carnica*, the commercial apiary located in Motobes region Kafr El-Sheikh Governorate, Egypt, during the active period of the summer season 2022. Each colony consisted of five combs (three brood combs, two honey, and stored pollen combs) covered with bees and headed by newly mated queen sisters.

### 2.2 Apitoxin collector device

Api collecting electric shock device CJ 401 (Chung-Jin Biotech Ltd., Ansan, Korea) consists of a digital control board, five bee venom collection frames, wire electrodes, battery, Input /Output Voltage: 12 VDC (timer ON: 3 s. and timer-OF: 6 s). Finally, collector Frames: 46 × 28 cm. Honeybees were subjected to a bee venom-collecting electric shock device. There were five parallel wires in the electrode-equipped Api collecting frames of the device. Every frame in a hive was put on top of the combs before being wired to an electro-stimulator. Electrical impulses stimulate bee workers to sting via a latex sheet that has been put on a glass plate of the device frame. Bees that came into touch with the wires were mildly shocked by the electricity and stung the glass surface. The other bees were agitated and mobilized as a result of the alarm odor that was released by the venom, and they began to sting as well. To ensure the safe transmission of the frames containing the fresh Api to the laboratory for 24–48 h, they were meticulously packaged inside a specialized container. The processing of dry Api scraping was implemented by a sharp scraper under laboratory conditions.

### 2.3 Chitosan bee’s extraction

Several phases were involved in the extraction of biopolymers of chitin and chitosan from a novel potential source, with dead corniolan honeybee hybrids were collected in front of bee hives during the autumn season of 2022 from the commercial apiary located in Motobes region Kafr El-Sheikh Governorate, Egypt. To extract chitin, the protein (deproteination) and mineral (demineralization) elements of sub-pestilence was first dissolved and removed. The raw honey bee *Apis mellifera* material was first ground using CM 190 Cemotec TM, Denmark. Demineralization was then performed using the Hackman technique with minor modifications (Haydarova and Ikhtiyarova, 2019), by treating the crushed raw material with 2 M hydrochloric acid for 5 h at 25–27 ^0^С. Then, deproteination was accomplished by treating the pulverized raw materials with a 1N sodium hydroxide solution for 1 h at a temperature of 80−85^0^С. Each step was preceded by a natural wash water reaction (pH = 7) of the raw components. Chitosan, a high molecular weight glucosamine polymer, was synthesized by deacetylation of chitin in the presence of a 35% aqueous NaOH solution at a temperature of 80−85^0^С for 4 h and dried at 60–65^0^С. The resultant mass was decolorized with 3% H_2_O_2_ and washed with 10% ethanol. The chitin content (WC %) was calculated according to Mol et al., (2018) using the following formula:


(1) $$\begin{array}{l} WC\%=\left(\mathrm{weight}\;\mathrm{of}\;\mathrm{the}\;\mathrm{raw}\;\mathrm{material}-\mathrm{weight}\;\mathrm{of}\;\mathrm{the}\;\mathrm{final}\;\mathrm{product}\right)\\ /\mathrm{weight}\;\mathrm{of}\;\mathrm{the}\;\mathrm{raw}\;\mathrm{material}\times 100 \end{array}$$

Each step was preceded by a natural wash water reaction (pH = 7) of the raw components. Bee chitosan was synthesized by deacetylation of chitin in the presence of a 35% aqueous NaOH solution at a temperature of 80–85°C for 4 h and dried at 60–65°C. The resultant mass was decolorized with 3% H_2_O_2_ and washed with 10% ethanol.

### 2.4 Physicochemical characterization

ChB was diluted in 2% (w/v) acetic acid and filtered to achieve the purification of bee chitosan. The polymer was precipitated by adding 1.0 M NaOH to the chitosan solution while stirring. The precipitated polymer was rinsed with distilled water until the filtrate pH matched the distilled water. The finished product was vacuum-dried in an oven at 60°C after repeated acetone washes. ChB samples were washed with distilled water to a neutral pH according to kaya et al. 2015 (Kaya et al., 2015). The yield (Y) of ChB was estimated by weighing dried chitosan and using the following equation.


(2) ChByield%=weight ofbeechitosan−weight ofbeechitin/100

Bruker Optics (Tensor, 47IFS), Germany, employed for FT-IR to characterize chitin and chitosan. The following formula was used to calculate the Degree of deacetylation (DD) of chitosan samples:


(3) $$DD\;\left(\%\right)=100-\left[\left(A1658/A3450\right)/1.33\times 100\right]$$

where the ratio A_1655_/A_3450_ for fully N-acetylated chitosan is indicated by the factor of 1.33 (Kaya et al., 2014). The following equations were used to calculate molecular weight (MW), ash (AC), water binding capacity (WBC), fat binding capacity (FBC), and moisture content (MC) of drying honey bee chitosan by vacuum oven for 24 h at 110°C (Sun et al., 2007; Rødde et al., 2008; Marei et al., 2016).


(4) $$\begin{array}{c} MW=\mathrm{weight}\;\mathrm{of}\;\mathrm{the}\;D-\mathrm{glucosamine}\;\mathrm{hydrochloride}\\ -\mathrm{weight}\;\mathrm{of}\;\mathrm{the}\;\mathrm{chitosan}\;\mathrm{oligomer}\times 215.5\; \end{array}$$

(5) $$\mathrm{AC}\%=\left(\mathrm{weight}\;\mathrm{of}\;\mathrm{the}\;sa\mathrm{mple}/\mathrm{weight}\;\mathrm{of}\;\mathrm{the}\;\mathrm{residue}\right)\times 100$$

(6) $$MC\%=\left(\begin{array}{l} \mathrm{weight}\;\mathrm{of}\;\mathrm{chiiosan}\;\mathrm{before}\;\mathrm{dryin},\;g,\\ -\mathrm{weight}\;\mathrm{of}\;\mathrm{chitosan}\;\mathrm{after}\;\mathrm{drying},\;g\\ /\mathrm{weight}\;\mathrm{of}\;\mathrm{chitosan}\;\mathrm{before}\;\mathrm{drying},g \end{array}\right)\times 100$$

(7) $$WBC\%=\left(\mathrm{water}\;\mathrm{bound},g\right)/\left(\mathrm{weight}\;\mathrm{of}\;\mathrm{sample},g\right)\times 100$$

(8) $$FBC\;\left(\%\right)=\left(\mathrm{fat}\;\mathrm{bound},g\right)/\left(\mathrm{weight}\;\mathrm{of}\;\mathrm{the}\;\mathrm{sample},g\right)\times 100$$

The following equation was used to calculate the chitosan crystallinity index (CrI) according to Zhang et al., (2000)


(9) CrI=_I110−Iam_∕I110

where I_am_ is the greatest intensity in the corresponding amorphous area at 2θ ≈ 11°, while I_110_ is the maximum intensity at 2θ ≈ 20°.

### 2.5 Preparation of ChB NPs

The ionic gelation process was used to create ChB NPs. Extracted chitosan from dead bees (ChB) solutions were prepared by dissolving 1 g of chitosan in 100 mL of 1.0% aqueous acetic acid and stirring until the liquid became translucent. Following that, NaOH (0.01 N) was used to increase the acidic pH until (pH 5.5) and facilitate the cross-linking reactions between chitosan and tripolyphosphate (TPP) ions. A ChB acidic solution was combined with a basic TPP solution (1.0%) at room temperature and stirred at 150 rmb to create the ChB NPs. TPP was added dropwise to the ChB solution to begin the spontaneous synthesis of ChB NPs. Then, the suspension was stirred under magnetic stirring at room temperature and left to qualify for 30 min. The ChB NPs were then centrifuged at 3000 rmb for 15 min at 3–5°C and freeze-dried with 10% (m/m) trehalose in a Freeze-dryer for 24 h (Arif et al., 2021). The nanoparticles loaded with bee venom were prepared by introducing venom at a concentration of 1 g/20 mL of deionized water and stirring for 10 min. Subsequently, the venom solution was combined with the TPP solution prior to the addition of the chitosan solution (Schematic 1A).

### 2.6 Distributions and characterizations of ChB NPs

The particle size and ζ-potential were estimated by DLS (Malvern Instruments, UK). 3 mL of bare ChB NPs and Api@ChB NPs were diluted in deionized water and placed in a cell cuvette. The size was estimated four times and then and three ζ-potential charges the mean ± SD (Sharaf et al., 2021). FTIR spectrometers evaluated functional groups in prepared samples. Potassium bromide-treated samples (KBr). A disk was pressurized and measured at 500–4,000 cm^−1^with a resolution of 4.0 cm^−148^. To Transmission electron microscopy (TEM) images of ChB and Api@ChB NPs, 1% phosphotungstic acid discolored the samples on copper grids (PTA). After air-drying at ambient temperature, materials were analyzed using (Philips 400; TEM, Netherlands) at 80 kV (Hou et al., 2021).

### 2.7 Antimicrobial susceptibility

#### 2.7.1 Bacterial and fungal sample collection

All the isolated Gram-positive bacteria *Staphylococcus aureus, Staphylococcus.hominis, and Enterococcus feacalis*, Gram-negative bacteria *Escherichia coli, Klebsiella pneumonia and Acinetobacter baumannii* and one unicellular fungal (*Candida auris*) and multicellular (*Aspergillus nigar)* were collected from the Microbiology Department, Faculty of Medicine, Cairo University, Egypt through the proper protocol and identified and diagnosed based on morphological characteristics and biochemical examinations according to the standard methods of diagnosis and confirmed with the Vitek 2 compact (Harrigan and McCance, 1976; Procop et al., 2017).

#### 2.7.2 Agar well assay

The conventional agar-well diffusion technique was used to investigate the antibacterial activity, according to Dipankar and Murugan (2012), the test specimens were subcultured into a pure culture in nutrient broth, and the strain was then evenly disseminated with Muller-Hinton agar (MHA) on sterile petri plates. A sterile cork-borer was used to drill a circular borehole in plates that were 9 mm in diameter. Hundred subcultured into a pure culture in nutrient broth, and the strain was then evenly disseminated with Muller-Hinton agar (MHA) on sterile petri plates. A sterile cork-borer wa_2_O were used as positive control and negative control, respectively). Antifungal activities of Api, ChB NPs, and Api@ChB NPs were performed on 5-day-old cultures of *Aspergillus niger* and *Candida auris* on Czapex Dox Agar (CDA) at 28 *C. auris* was obtained by gently scraping fungal lawns with three to 4 mL of sterile normal saline. Hundred μl of this liquid spore suspension was equally dispersed over new Potato Dextrose Agar (PDA) plates. A sterile cork-borer created a 6 mm circular well in plates. To test antifungal activity, each sample was added to the well, filling it with 100 μa, and then incubated for 1–5 days at 28 ng. The zones of inhibition were determined.

#### 2.7.3 Determination of minimum inhibitory concentration and minimum lethal concentration

By using the usual dilution approach, a broth microdilution assay was used to estimate the MIC of antibacterial activity in 96 multi-well microtiter plates (CLSI M07-A8) (Hayato et al., 2012) 0.100 μL of TSB (Himedia) was added evenly across all wells. A volume of 100 μL from each Api, ChB NPs, and Api@ChB NPs (–2.5 μg mL^−1^) was pipetted into the wells of the first row of the microtiter plate. Finally, 100 μL of freshly made, 0.5 McFarland matching turbid bacterial solution was put into each well. Each plate contained two columns to serve as both positive and negative controls. Wrapped plates were incubated for 18–24 h at 37°C. The plates were visually inspected for the presence or absence of turbidity against a dark background. The MIC was determined as the lowest concentration at which there was no discernible bacterial growth when compared to controls.

Additionally, stock inoculum suspensions were made in trek diagnostic systems sterile saline with 1% tween 80 from 7 days colonies on potato dextrose agar slants (provided by Remel, Lenexa, Kans) used to estimate the MIC of antifungal activities. A 95% of the stock inoculum suspensions measured 0.9 × 10 (Mezzana, 2008) to 4.5 × 10 (Mezzana, 2008) CFU/mL. On test day, each microdilution well was infected with 100 μL of the diluted (Twofold) conidial inoculum suspensions in liquid potato. Then, 200 μL per well of Dextrose Agar (PDA) and microdilution trays were tested after 4 days at 28°C. The MIC goals were the lowest Api, ChB NPs, and Api@ChB NPs concentrations that inhibited growth completely (100% inhibition). By sub-culturing 20 μL from the clear wells of the MIC, MLC was ascertained.

### 2.8 Determination of cytotoxicity study anticancer properties using MTT assay

#### 2.8.1 Cytotoxicity

MTT was used to determine the cytotoxicity of normal Vero ATCC CCL-81 cell line, Caco2 ATCC ATP-37, and HepG2 ATCC HB-8065 cancer cells (Berridge and Tan, 1993). The 96-well tissue culture plate was injected with 1 × 10 (Chen et al., 1998) cells/mL (100 μL well) and cultured at 37°C for 24 h to form a full monolayer sheet. After a confluent cell sheet had been developed, the growth medium was decanted from 96-well microtiter plates, and the cell monolayer was washed twice with wash media. Api, ChB NPs, and Api@ChB NPs were diluted twice in the RPMI medium with 2% serum (maintenance medium). 0.1 ml of each dilution was tested in distinct wells, keeping three wells as controls with maintenance media at 37°C. Symptoms of toxicity were checked for monolayer loss, rounding, shrinkage, and cell granulation. MTT (5 mg mL^−1^ PBS) was prepared. In each well was added 20 μL of MTT. Then, shakeen at 150 rpm for 5 min to mix MTT into the medium. For MTT metabolization, the mixture was incubated for 4 h at 37°C and 5% CO_2_ (If needed, wipe the plate with paper towels). Resuspend formazan (MTT metabolic mixure) in 200 μL DMSO. On a shaking table, mix the formazan and solvent for five min at 150 rpm at OD560 nm (Wang et al., 2017). There OD should be a direct correlation between cell amount and optical density.


$$\%\mathrm{Cytotoxicity}=\left(OD\;\mathrm{test}-OD\;\mathrm{blank}\right)/\left(OD\;\mathrm{control}-OD\;\mathrm{blank}\right)$$

where OD optical density test means the cells exposed to the Api, ChB NPs, and Api@ChB NPs sample, control means the control sample, and blank means the wells without normal Vero ATCC CCL-81 cell line, Caco2 ATCC ATP-37, and HepG2 ATCC HB-8065 cancer cells.

#### 2.8.2 Morphological analysis of cell viability

The cellular morphology was examined using an inverted microscope. After 48 h of exposure to a variety of Api, ChB NPs, and Api@ChB NPs concentrations or as compared to the control, the morphology of normal Vero ATCC CCL-81 cell line, Caco2 ATCC ATP-37, and HepG2 ATCC HB-8065 cancer cells was analyzed for alterations and images were taken.

### 2.9 Statistical analysis

The One Way ANOVA test was used to compare the arithmetic averages of the inhibition regions to the quantities of the different compounds whose biological inhibitory activity was assessed for bacterial species using Duncan’s polynomial test at *p < 0.05 (Gerber and Voelkl, 2012).

## 3 Results and discussion

### 3.1 Physiochemical analyses of ChB

Dead honeybees yielded 25% chitin. The physiochemical analyses of bee chitosan (ChB) showed the yield of chitosan (Y) from the chitin was 45.1 ± 2.5%, possibly due to the source and during deacetylation and precipitation process to remove each of impurities and proteins (Amor et al., 2023). ChB had a DD and WBC values of 79.15 ± 0.7% and 622.7 ± 5.8%, respectively. WBC in ChB was observed to fall within a narrower range (458–805%) than that previously reported by Marei et al. (2016). The FBC value of ChB was 399.5 ± 13.3%. The findings of this study indicate that the FBC of the ChB was comparable to the values reported by Cho et al. (1998) and No et al. (2000), ranging from 217 to 403%.

When chitosan is heated in the presence of air, it totally decomposes, leaving behind the inorganic residue known as ash content (AC). ChB AC concentration indicates calcium carbonate removal and demineralization efficiency. High-quality ChB has less than 1% AC (Nessa et al., 2010). In accordance with Table 1. ChB was found to have the highest AC and MC, coming in at 0.7 ± 0.1% and 9.85 ± 0.3%, respectively. These results indicated that ChB had the highest quality. In addition, the produced ChB has a MW of 20 kDa, which is consistent with a previous study by Sahebzadeh et al. (2024). According to Khan et al. (2000) mentioned that the molecular weight of chitosan samples decreased with increasing alkali treatment times. The alkali treatment resulted in a greater abundance of amine (–NH_2_) groups and a reduced abundance of acetyl amide (− NHCOCH_3_) groups (Khan et al., 2000). Chitosan’s molecular weight might be reduced by depolymerization in response to the increased alkalinity (Chebotok et al., 2006).

**Table 1:** Zone diameter (mm) interpretative standards chart and tested samples for the well diffusion method of determining antibiotic sensitivity and resistance status of Common Human Bacterial, Fungal Pathogens.

**Tested microorganisms**	Samples			Control		
	Api	ChB NPs	Api@ChB NPs	Negative (Water)	Positive (Fluconazole)	Positive (Gentamicin)
Gram-positive						
*E. faecalis*	14.6 ± 0.3^b^	17.8 ± 0.2^c^	21.4 ± 0.^3d^	*NI	12 ± 0.1^a^	-
*S. aureus*	9.5 ± 0.7^a^	13.4 ± 0.3^c^	16.6 ± 0.7^d^	*NI	13 ± 1.4^b^	-
*S. hominis*	11.6 ± 0.7^b^	15.1 ± 0.6^c^	19.9 ± 1.3^d^	*NI	11 ± 1.^73a^	-
Gram-negative						
*E. coli*	13.4 ± 0.3^b^	17.7 ± 0.6^c^	23.1 ± 1.5^d^	*NI	12.1 ± 0.8^a^	-
*K. pneumonia*	6.1 ± 1.5^a^	11.4 ± 0.8^b^	14.2 ± 1.6^c^	*NI	15 ± 1.2^d^	-
*A. baumannii*	15.2 ± 1.6^b^	17.3 ± 0.1^c^	22.1 ± 1.8^d^	*NI	14.5 ± 2.3^a^	-
Fungi						
*C. auris*	7.4 ± 0.8^a^	8.2 ± 1.6^b^	12.1 ± 0.6^c^	*NI	-	13 ± 0.7^d^
*A. niger*	7.1 ± 0.^6a^	15.9 ± 1.3^b^	22.2 ± 1.6^c^	*NI	-	17 ± 1.9^d^

Numerical data are reported as mean ± SD (*n* = 3). Values in each column followed by different letters are significantly different a,b, in Duncan test (*P* < 0.05); statically differences between positive control and samples in Duncan test. *NI, no inhibition.

**Figure 1:**
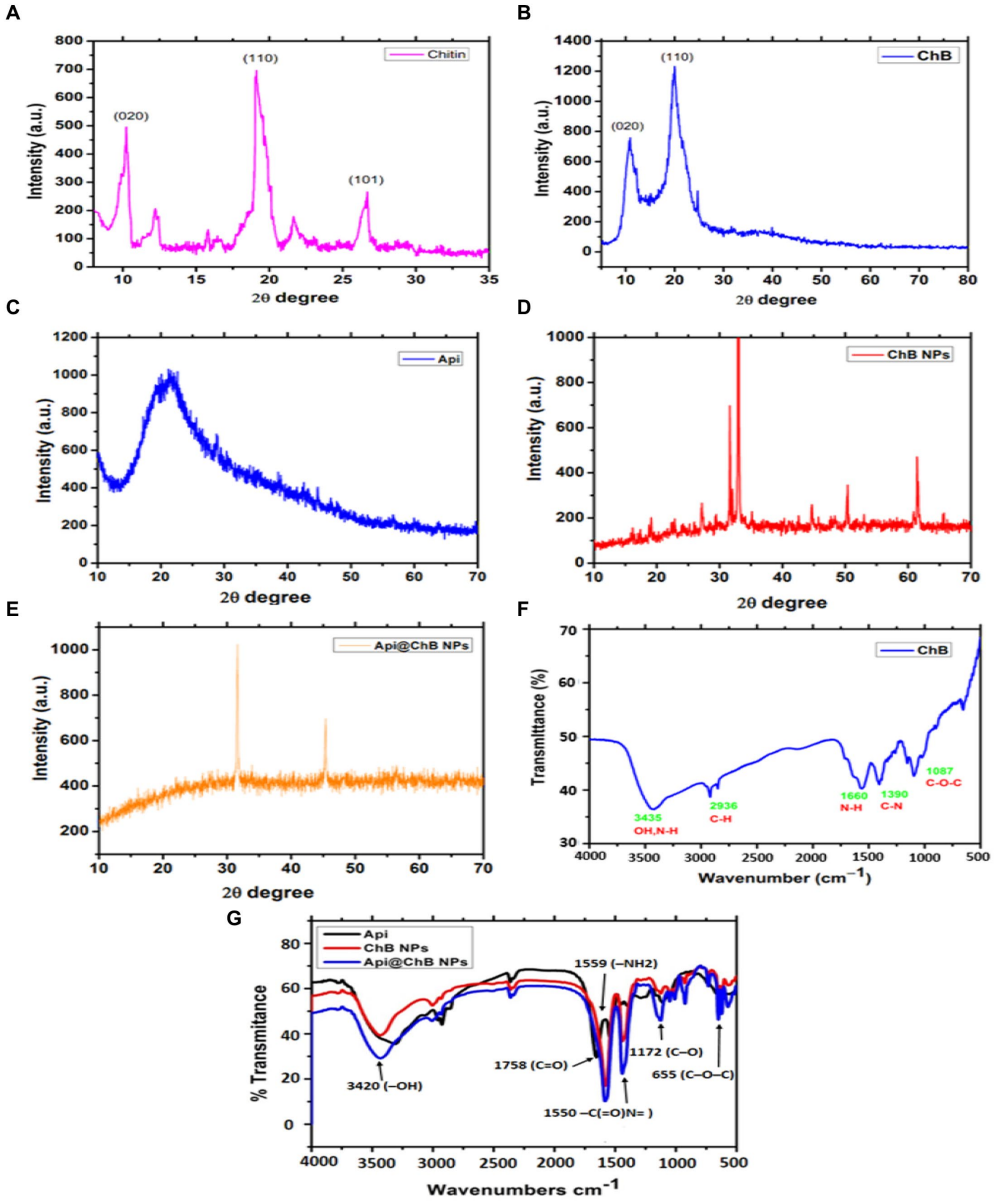
XRD patterns of extracted bees *Apis mellifera* **(A)** Chitin; and **(B)** honey bee chitosan (ChB), **(C)** Api; **(D)** ChB NPs; and **(E)** Api@ChB NPs; **(F)** FTIR spectra of extracted bees *Apis mellifera* pure chitosan and **(G)** FTIR spectra of Api, ChB NPs, and Api@ChB NPs.

X-ray diffraction by a crystalline structure in the sample allows the study of atomic and molecular structure of the crystal. The XRD patterns of honey bee chitin and chitosan samples were located at angles (60 min) at a 2-theta-scale are shown in Figure 1.

*Apis mellifera* chitin samples showed significant strong peak at 2θ = 9.9°C (020), 2θ =19.8°C (Sangboonruang et al., 2020), 2θ =26.5°C (Elnosary et al., 2023), and minor peaks at 12.1°C, 22.1°C. XRD results of chitin yielded by crab, shrimp, krill, and insects such as *Geolycosa vultuosa* and *Sitophilus granarius* are similar to the peak indicated by this study (Yen et al., 2009; Sajomsang, 2010; Liu et al., 2012; Wang et al., 2013; Kaya et al., 2014; Figure 1A). The high crystallinity of pure ChB was 82.2 ± 0.8%. The ordered crystalline structure of ChB is evidenced by the presence of two diffraction peaks at 2θ =10.5^0^ (020) and 2θ =20.2°0 (Sangboonruang et al., 2020), as shown in the XRD pattern of ChB in Figure 1B. Comparatively, the CrI value of chitosan isolated from different insects, such as beetles, cuttlefish, shrimp, and silkworms (*B. mori*), varied from 36 to 95% (Zhang et al., 2000; Paulino et al., 2006; Kaur et al., 2013; Morsy et al., 2019; Mohan et al., 2020).

Furthermore, FTIR spectra of the functional groups of pure ChB with strong peaks were observed at 3454 cm^−1^ and referred to –OH group stretching, peaks at 2936 cm^−1^ indicated carbonyl C–H stretching, a peak at 1087 indicated cm^−1^ C–O–C stretching, and peaks at 1390 to 1,660 cm^−1^ were attributed to a protonated amino (–NH_2_) group N-H bending vibration and a group C–N bending vibration (see Figure 1F).

### 3.2 Characterization of ChB NPs and Api@ChB NPs

#### 3.2.1 XRD patterns

XRD patterns for pure Api, ChB NPs, and Api@ChB NPs are shown in Figures 1A–C. The XRD patterns of pure Api had one wide peak, indicating that it represented a semicrystalline polymer (see Figure 1C). One peak was seen in the diffraction graph at a 2-theta-scale of 2θ = 20.13^0^ (020), and it could even be seen expanded to a 2-theta-scale of 2θ = 25.32^0^ (120). These findings concurred with those of Qiao et al. (2007). Figure 1D shows that the XRD patterns of ChB NPs were not very similar to those of ChB alone. Generally, synthetic polymers are recognized as amorphous materials (Wang and Mano, 2006; Yan et al., 2009). Thus, the regular arrangement of atoms and molecules produces sharp diffraction peaks, but amorphous regions result in broad halos. It is noticeable in Figure 1E that the peak at a 2-theta-scale of 2θ = 25.32^0^ disappeared in the Api@ChB NPs, suggesting that the Api was either in an amorphous state or in a state of molecular dispersion and completely encapsulated inside the polymer. Additionally, the decrease of Api in the crystalline state in the Api@ChB NPs indicated that interactions may have taken place between the drug and copolymer in the hydrogels. The ChB NPs were semisolids, and the Api was a powder at room temperature. These results were consistent with those of the literature (Hao et al., 2013; Jana et al., 2015; Thamilarasan et al., 2018; Badawy et al., 2019).

#### 3.2.2 FTIR analysis

FTIR spectroscopy was used to investigate the functional groups of Api, ChB NPs, and Api@ChB NPs (Figure 1G). The Api had two characteristic regions, 1,600 to 1,700 cm^−1^ and 1,500 to 1,550 cm^−1^, in its spectrum unique to its protein secondary structures, called amide I and II bands –C(=O)N = stretching. In the FTIR spectrum of ChB NPs, a strong peak was observed at 1,758 cm^−1^ (carbonyl –C=O stretching), as well as a peak at 1091 to 1,172 cm^−1^ (C–O stretching) and P=O stretching (Gylienë et al., 2003; Zvezdova, 2010). The observed outcomes have been ascribed to the association between phosphoric and ammonium ions. In light of the evidence presented, it may be inferred that the tripolyphosphoric groups of TPP are chemically bonded to the ammonium groups of chitosan. The inter-molecular and intra-molecular interactions are intensified in chitosan nanoparticles (Mohammadpour Dounighi et al., 2012). In the FTIR spectrum of Api@ChB NPs, the –OH group of stretching vibrations caused a peak at 3420 cm^−1^ for the primary functional group of the chitosan. The absorption peaks at 1559 and 1,405 cm^−1^ were attributed to a protonated amino (–NH_2_) group N–H bending vibration and an alkyl group C–H bending vibration, respectively. The absorption peaks at 1096 and 655 cm^−1^ were attributed to the glucopyranose ring in the chitosan matrix caused by the antisymmetric stretching vibration of C–O–C bridges. This result was consistent with data in the literature (Zhang et al., 2011; Liu et al., 2013), and it suggested that the Api was successfully encapsulated in the ChB NPs.

### 3.3 Distribution of ChB NPs and Api@ChB NPs

#### 3.3.1 DLS

**Figure 2:**
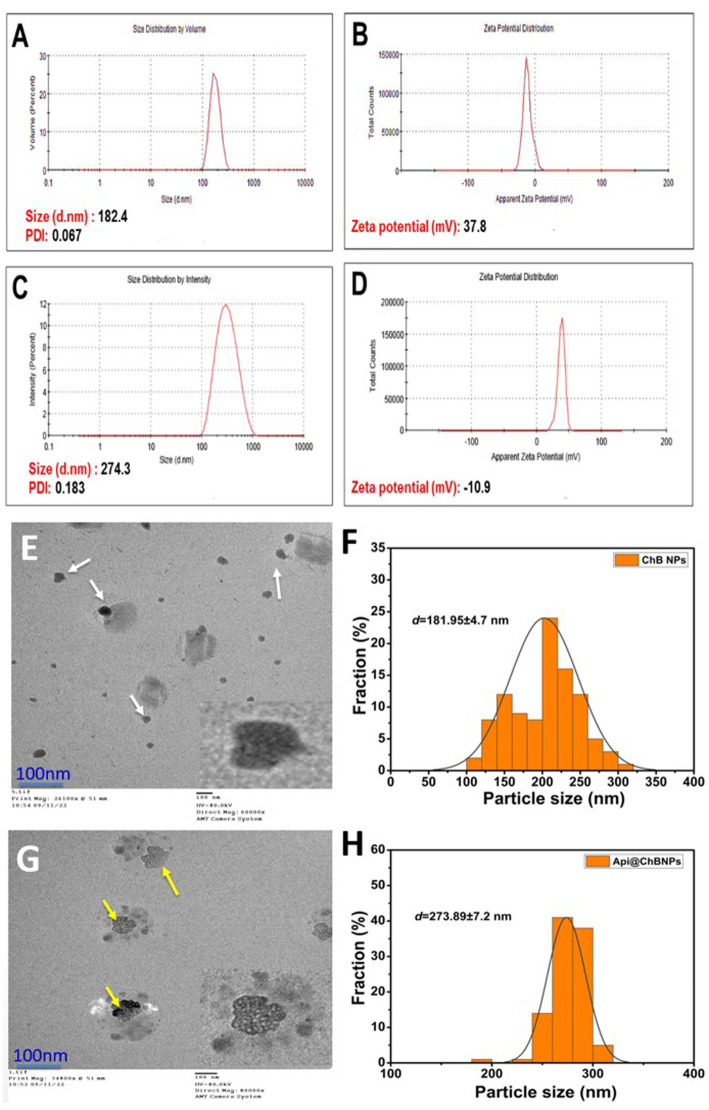
Hydrodynamic size, polydispersity index (PDI), and ζ-potential **(A,B)** ChB NPs; and **(C,D)** Api@ChB NPs. The numbers are given as mean standard deviation (SD) for ζ-potential (*n* = 3) and for particle size and PDI (*n* = 4). Average particle size and data size distribution of prepared samples **(E,F)** ChB NPs and **(G,H)** Api@ChB NPs measured by TEM (scale bar = 100 nm).

Dynamic light scattering (DLS) was used to measure the particle size (PS), polydispersity index (PDI), and ζ-potential of ChB NPs and Api@ChB NPs. All of the formulas’ average results revealed a PS distribution in nanometers (Figure 2). DLS findings showed that the ChB NPs (see Figure 2A) and Api@ChB NPs (see Figure 2C) had sizes of approximately 182 ± 2.1 nm and 274 ± 3.8 nm, respectively. The findings demonstrated that the addition of bee venom increased the size of the ChB NPs. The ability of nanochitosan particles to successfully load Api may be shown by the increased size of loaded ChB NPs carrying Api relative to those without Api (Soares et al., 2012). Nanocomposites are often made for effective biomedical applications in a size range of 20 to 300 nm in attempts to produce the smallest particles that might spread to any area of the body (Krug and Wick, 2011). According to Alalawy et al. (2020), chitosan nanoparticles had a PS range of 92.1 to 157.3 nm, while Api@ChB NPs had a PS range of 147.3 to 269.6 nm. Furthermore, the PDIs for ChB NPs and Api@ChB NPs were 0.183 and 0.067, respectively (see Figures 2A,C). However, a particle with a very narrow distribution has PDI values between 0.01 and 0.30, which is the optimal size for dispersion stability and homogeneity (PDI < 0.5). Their ζ-potential values generally predict the stability of nanoparticles; here, the determined ζ-potential values were 37.8 ± 1.2 mV and − 10.9 mV for ChB NPs and Api@ChB NPs, respectively (see Figures 2B,D). Electrostatic balancing stabilizes the ζ-potential value above 30 mV and below −30 mV. However, an increase in the positive ζ-potential charge may have been due to the ionization of the amino functional groups (–NH_2_) in the capping moieties at an acidic pH, which formed a repulsive barrier that prevented aggregation and improved the colloidal stability of the ChB NPs (Zhang et al., 2000). Furthermore, our findings indicated that venom loading reduced the particle’s zeta potential of Api@ChB NPs. This decrease may be caused by electrostatic contact between carboxyl groups on the venom molecule’s surface and amine groups at certain places on the chitosan molecule. However, the attachment of venom molecules did not effectively suppress all of the positive surface charge of chitosan molecules. It seems that a large fraction of free amine groups on the chitosan chain remained unoccupied, and this confirmed that Api had been successfully loaded into the nanoparticles. Similar results were obtained by Taher et al. (2017). Mohammadpour Dounighi et al. (2012) reported a reduction in the zeta potential of chitosan nanoparticles subsequent to the loading of *Mesobuthus eupeus* scorpion venom.

#### 3.3.2 Surface morphology using TEM analysis

The surface morphological structure was investigated further using TEM. Figure 2 shows TEM images of ChB NPs and Api@ChB NPs at a low magnification scale of 100 nm. TEM analysis revealed the existence of two distinct populations of particles in terms of size, small and big particles ranging from around 100 to 330 nm (Figures 2E,G). ChB NPs without Api had an amorphous shape and were evenly distributed. Additionally, some particles were shown to be in an agglomerated state with some aggregation. They had an average size of 181.95 ± 4.7 nm (see the white arrows in Figure 2A) and the mean length of size distributions (see Figure 2F). The Api@ChB NPs were larger than the ChB NPs without Api, had an excellent dispersion of 273.89 ± 7.2 nm, and were surrounded by a light layer. This might have been because the Api was entrapped outside the carbohydrate polymer matrix of the chitosan (see the white dots and yellow arrows in Figure 2G). The layer may have been responsible for the increase in the hydrodynamic size of the Api@ChB NPs over the ChB NPs without Api. The increased size of the Api@ChB NPs was related to the enhanced efficiency of the Api and its assimilation into the ChB NPs. Figure 2H depicts the mean diameter of particle size distribution. These results were in agreement with those of Taher et al. (2017) and El-Didamony et al. (2022). It was discovered that the chitosan nanoparticles had a spherical form and that there was a layer around the center of the nanoparticles that included nanoparticles loaded with bee venom. This finding agreed with that of Gan and Wang (2007), who reported that the large size may be due to venom breakdown in the TTP solution at the time of cross-linked nanoparticle formation, with these protein molecules confined inside the polymeric framework of chitosan nanoparticles.

### 3.4 Antimicrobial activity

#### 3.4.1 Agar well diffusion assay

**Figure 3:**
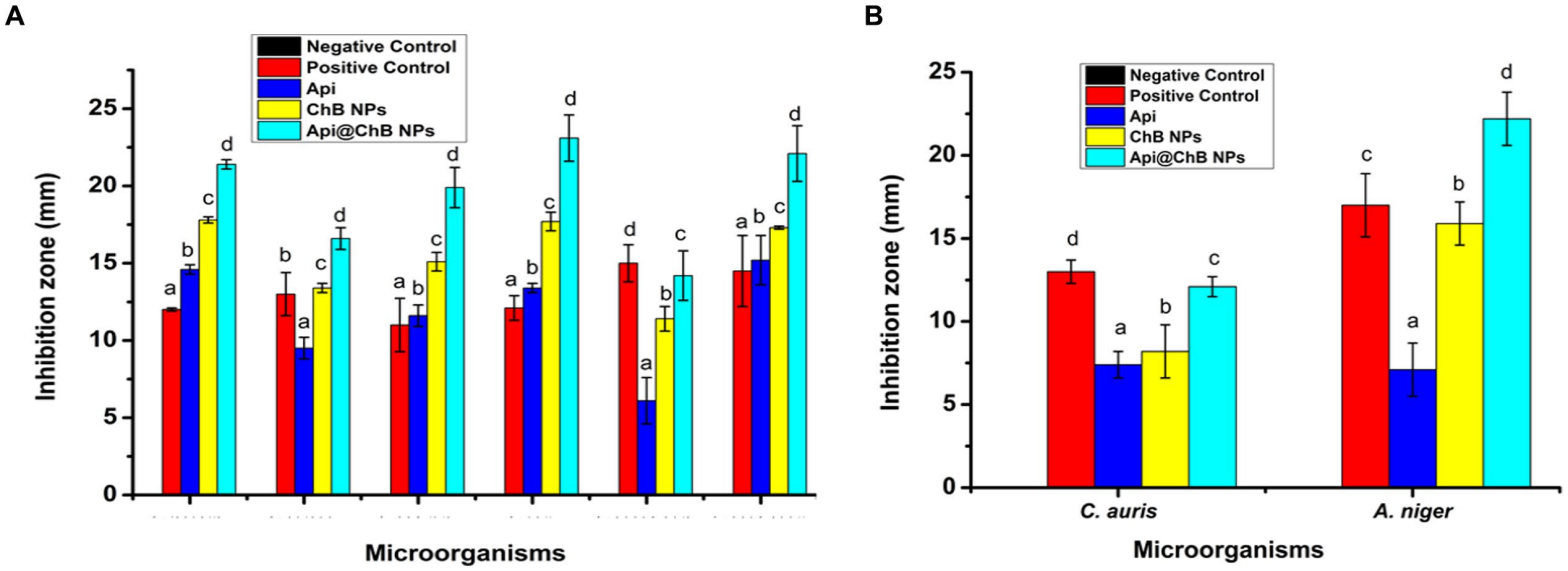
The inhibition zone **(A)** different pathogenic bacteria strains *E. faecalis, S. aureus*, *S. hominis, E. coli, K. pneumonia*, and *A. baumannii*. **(B)** *C. auris* and *A. niger* against by Api, ChB NPs, and Api@ChB NPs. Values in each column followed by different letters are significantly different ^a,b,^ in Duncan test (*P* < 0.05); statically differences between positive control and tested samples using one-way ANOVA.

The Api, ChB NPs, and Api@ChB NPs were tested for their antibacterial properties by measuring the length of the inhibitory zone (in millimeters) that formed on agar plates during the incubation time. These findings are broken down into parts and detailed in Table 1, Figure 3, and Supplementary Figure S3. A negative control was formed by filling a standard well with 100 μL of untreated pure water and observing whether or not any inhibitory zones emerged with any of the microbial strains. Thus, pure water did not influence nanoparticle activity. Gentamicin (MIC 8 μg mL^−1^) was selected as a standard antimicrobial agent; its inhibition zones ranged from 15 ± 1.2 to 30 ± 2.3 mm, as illustrated in Table 1 and Supplementary Figure S1.

The inhibition zone is depicted in Figure 3A and Supplementary Figure S3. The figures show that the Api had a stronger effect on the Gram-negative strain *Acinetobacter baumannii,* with an inhibition zone of 15.2 mm, than on the Gram-positive strains *Staphylococcus hominis* (inhibition zone of 11.6 ± 0.7 mm) *and E. faecalis* (inhibition zone of 14.6 ± 0.3 mm). *K. pneumoniae* and *S. aureus* had significantly smaller inhibition zones of 6.1 ± 1.5 mm and 9.5 ± 0.7 mm, respectively; this was due to the melittin in the honeybee venom, which is more effective against Gram-positive bacteria than Gram-negative bacteria. Most of the bee venom molecules are made up of the peptide melittin (40–48%, w/w), which has a potent cytolytic and antibacterial impact against numerous bacterial strains (Zolfagharian et al., 2016). This results of the antimicrobial activity are not contrasted with those reported by Elnosary et al. (2023). Bee venom has previously been shown to damage the integrity of the bacterial outer membrane by creating holes that disintegrate the bacterial cell. This mechanism is essential for healing and survival during a bacterial infection (Malanovic and Lohner, 2016; Khalil et al., 2021). El-Bahnasy et al. (2022) and Elnosary et al. (2023) found that increased Api levels were effective against Gram-negative and Gram-positive organisms. Melittin and phospholipase A2 (PLA2), which impact cell membrane permeability, maybe the major antibacterial components of Api. Melittin forms holes in phospholipid bilayers, breaking down phospholipid groups or releasing Ca^++^ (Wu et al., 2016), and PLA2 hydrolyzes phospholipids, indirectly damaging the bacterial cell membrane (Banks and Shipolini, 1986) (Schematic 1B).

The most effective of our nanoformulations in terms of inhibition zones (with some minor differences) were ChB NPs, followed by Api@ChB NPs. It is interesting to note that the ChB NPs exhibited good microbial activity compared with the Api@ChB NPs against all of the tested strains. The ChB NPs yielded inhibition zones of 11.4 ± 0.8 to 17.8 ± 0.2 mm. However, the Api@ChB NPs yielded significantly larger inhibition zones of 14.2 ± 1.6 to 23.1 ± 1.5 mm. In general, our results showed a more synergistic effect of the Api@ChB NPs on Gram-negative strains than Gram-positive strains, and this signified that the effect of this combination was greater than the sum of the individual effects of the Api and the ChB NPs (Bassolé et al., 2010). Nanopolymers, particularly nanochitosan, were among the most studied nanoparticles due to their bioactivity and usefulness as drug transporters and antimicrobial, antitumor, and gene delivery agents, either alone or in conjunction with other active substances (Gan and Wang, 2007; El Rabey et al., 2019). Several previous studies reported the same finding of unmodified chitosan being more effective against Gram-negative than Gram-positive strains (Eaton et al., 2008; Silva et al., 2010; Elnosary et al., 2023).

### 3.5 Antifungal activity

One unicellular fungal (*Candida auris*) and multicellular (*Aspergillus nigar)* isolates were identified using the VITEK2 system. It showed that these fungi had confidence intervals of 96% (excellent) and 95% (very good). Fluconazole, an antifungal used as a positive control (see Supplementary Figure S2), produced inhibition zones of 13 ± 0.7 mm for *A. nigar* and 17 ± 1.9 mm for *C. auris* (see Table 1, Figure 3, and Supplementary Figure S3). The Api, ChB NPs, and Api@ChB NPs were used at a concentration of 100 μg mL^−1^ against these two fungi. It was found that all three substances had antifungal properties. The Api produced inhibition zones of 7.4 ± 0.8 and 7.1 ± 0.6 mm for *C. auris* and *A. nigar*, respectively. The ChB NPs produced inhibition zones of 8.2 ± 1.6 and 15.9 ± 1.3 mm for *C. auris* and *A. nigar*, respectively. However, the Api@ChB NPs were the most effective against these fungi, producing inhibition zones of 12.1 ± 0.6 and 22.2 ± 1.6 mm for *C. auris* and *A. nigar*, respectively. The results are shown in (Table 1, Figure 3B, and Supplementary Figure S3a). It is interesting that the Api and nanoformulations were effective and could inhibit the growth of *A. nigar* even after the fungus had been cultured for 5 days. The color of the *A. nigar* changed from white to black (see Figure 3B and Supplementary Figure S3b). Our results confirmed that the use of bee products in the treatment of infectious diseases caused by *Candida* is extremely restricted (Lee, 2016). This may be due to the antioxidants in Api, which may regulate the generation of reactive oxygen species (ROSs) in fungi, followed by damage to the fungus’s lipids, proteins, and DNA; cell death may occur when this balance of components is upset inside the fungal cell (Ratajczak et al., 2021). El-Didamony et al. (2022) found that bee venom had a substantial inhibiting impact against *C. albicans* ATCC 90023, *Candida neoformans*, and *Kodamaea ohmeri*, and they attributed this effect to the regulation of ROS generation by oxidative stress. Similar results showed that bee venom had antifungal efficacy against 10 clinical strains of *C. albicans,* producing MIC values of between 62.5 and 125 μg mL^-1102^. Another study showed antifungal activity of bee venom melittin against the fungal yeasts *C. albicans, C. parapsilosis, C. tropicalis,* and *C. krusei*, with MIC values of between 30 and 300 μg mL^-1105^.

### 3.6 MIC_S_ and MLC assays

The broth dilution technique was used to determine the bacteriostatic effects of Api, ChB NPs, and Api@ChB NPs against various harmful bacteria (Selim et al., 2020). As shown in Table 2, ChB NPs outperformed Api in antimicrobial assays against Gram-negative and Gram-positive bacteria. At low concentrations, ChB NPs were highly antimicrobial. However, adding Api increased the activity significantly. The visual turbidity test showed that Api@ChB NPs inhibited *E. faecalis* at 12.5 μg mL^−1^; *S. hominis*, *A. baumannii*, and *E. coli* at 25 μg mL^−1^; and *S. aureus* and *K. pneumoniae* at 50 μg mL^−1^. For the comparable bacterial strain, control inhibition effectiveness varied from 8 μg mL^-1107^.

**Table 2:** MIC determinations of the NPs against fungal and bacterial human pathogens micro-strains.

Tested microorganisms	Samples					
	Api		ChB NPs		Api@ChB NPs	
	MICs	MLCs	MICs	MLCs	MICs	MLCs
Gram-positive bacteria						
*E. faecalis*	30	60	25	50	12.5	25
*S. aureus*	>100	>100	100	100	50	100
*S. hominis*	60	>100	50	100	25	50
Gram-negative bacteria						
*E. coli*	>100	>100	50	100	25	50
*K. pneumonia*	>400	>400	100	200	50	100
*A. baumannii*	60	>100	50	100	25	50
Fungi						
*Candida auris*	>100	>100	100	100	50	100
*Aspergillus niger*	60	>100	50	100	6.25	12.5

The second-order derivative of ChB NPs can show how a double concentration hinders these microorganisms’ microbial growth; it ranged from 25 μg mL^−1^ for *E. faecalis* to 50 μg mL^−1^ for *S. hominis*, *A. baumannii,* and *E. coli*, to 100 μg mL^−1^ for *S. aureus* and *K. pneumoniae*. Like previous studies, we proved that the killing efficiency of Api@ChB NPs with these bacterial strains was mostly less than that of the standard antibiotic gentamicin. Interestingly, ChB NPs had a statistically higher inhibition efficiency against Gram-positive bacteria at low concentrations than the other bacterial strains. In contrast, all the tested bacteria were strongly resistant to Api at concentrations of 100 μg mL^−1^ or more. These findings were similar to those of Issam et al. (2015), who reported that bee venom and melittin exhibited a broad spectrum of antibacterial activity against 51 strains of Gram-positive and Gram-negative bacteria. They had strong activity against even MRSA and vancomycin-resistant enterococci (VRE), with MIC values of between 6 and 800 μg mL^−1^. Thus, the MIC results revealed that Api@ChB NPs are more potent against Gram-negative and Gram-positive bacteria than other nanosubstances. Generally, the MIC values for Api@ChB NPs were lower than those for ChB NPs, suggesting that the entrapment process impacted the effectiveness of bee venom. This finding may be connected to the following theory. Because the examined microorganisms were only exposed to the experimental ChB NP concentrations once at the beginning of the experiment, some of these concentrations may have become tolerated by the time the experiment ended, resulting in relatively high MIC values. However, the Api@ChB NPs were available in moderate numbers throughout the experiment, and this may have resulted in decreased MIC values because the microbes may have developed tolerance to them. Prior studies have corroborated this theory (Sahoo et al., 2007; El Rabey et al., 2019).

### 3.7 Cytotoxicity against and morphological features of normal Vero ATCC CCL-81 cells, Caco2 ATCC ATP-37, and HepG2 ATCC HB-8065 cancer cells

**Figure 4:**
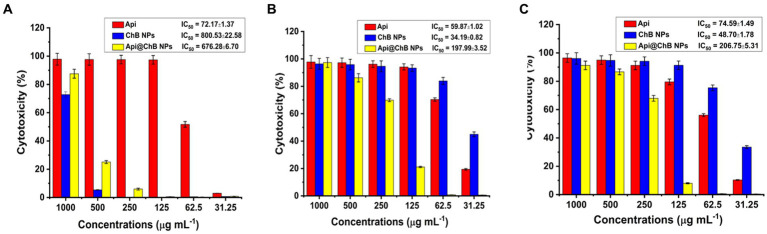
Cytotoxicity against Api, ChB NPs, and Api@ChB **(A)** normal Vero ATCC CCL-81 cells, **(B)** Caco2 ATCC ATP-37, and **(C)** HepG2 ATCC HB-8065 cancer cells. The numbers are given as mean standard deviation (SD) (*n* = 3).

Api, ChB NPs, and Api@ChB NPs were tested against Vero ATCC CCL-81 *Cercopithecus aethiops* normal kidney cells, Caco2 ATCC ATP-37 human colon cancer cells, and HepG2 ATCC HB human liver cancer cells at concentrations of 31.25–1,000 μg mL^−1^ to determine their ability to inhibit cancer cell growth. The test was conducted for 24 h at 37°C. There were three replicates for each concentration and the untreated control sample (Figures 4A–C). The severity of the toxicological impact was calculated according to the growth inhibition rate for the Api, ChB NPs, and Api@ChB NPs in relation to the control group, which grew at 100%.

The normal Vero ATCC CCL-81 cells were selected in order to evaluate the potential of Api, ChB NPs, and Api@ChB NPs as cytotoxic agents. Overall, all the tested concentrations showed cytocompatibility at concentrations of up to at least 62.5 μg mL^˗1^ (51.74%) of Api, 500 μg mL^˗1^ (94.60%) of ChB NPs, and 500 μg mL^˗1^ (>74.89%) of Api@ChB NPs after 24 h of incubation and IC50 values were 72.17, 800.53, and 676.28 μg mL^−1^ for the Api, ChB NPs, and Api@ChB NPs, respectively (Figure 4A).

The cancer Caco2 ATCC ATP-37 and HepG2 ATCC HB-8065 cells were selected in order to evaluate the potential of Api, ChB NPs, and Api@ChB NPs as anticancer agents. For Caco2 ATCC ATP-37 cells after 24 h incubation, all the tested concentrations showed cytocompatibility at concentrations of up to at least 62.5 μg mL^˗1^ (70.35%) of Api, 31.25 μg mL^˗1^ (83.93%) of ChB NPs, and 250 μg mL^−1^ (69.87%) of Api@ChBNPs and IC_50_ values were 59.87, 34.19, and 197.99 μg mL^−1^ for the Api, ChB NPs, and Api@ChB NPs, respectively (Figure 4B). For HepG2 ATCC HB-8065 cells after 24 h incubation, all the tested concentrations showed cytocompatibility at concentrations of up to at least 125 μg mL^−1^ (79.56%) of Api, 62.5 μg mL^−1^ (75.40%) of ChB NPs, and further evaluation of the Api@ChB NPs showed the lowest level of cytotoxic activity against at a concentration of less than 250 μg mL^−1^ (67.98%) and IC_50_ values were 74.59, 48.7, and 206.75 μg mL^−1^ for the Api, ChB NPs, and Api@ChB NPs, respectively (Figure 4C).

This finding corresponded well with the findings of Taher et al. (2017), who reported that the size of bee venom–loaded nanoparticles prepared with a concentration of chitosan of 1 mg/ml had good anticancer properties. Previous studies have also reported that most insect venoms in human stings contain various chemical combinations. They include amino acids, peptides, proteins, enzymes, carbohydrates, biogenic amines, volatile compounds, phospholipids, and pheromones (Carpena et al., 2020; Sangboonruang et al., 2020).

Furthermore, the morphological features of normal Vero ATCC CCL-81 cell lines and Caco2 ATCC ATP-37 and HepG2 ATCC HB-8065 cancer cell lines treated with different concentrations of Api, ChB NPs, and Api@ChB NPs were reported (Supplementary Figures S4–S6) and compared with those of the untreated cells. The 3T3 Phototox software estimated the concentrations of prepared samples in different cell lines using the absorbance values disclosed following the capture of the red dye and the relevant amounts of the Api, ChB NPs, and Api@ChB NPs employed in the viability studies. After the entry of chitosan nanoparticle-loaded Api into the cellular environment, a direct impact on the nucleus was seen, leading to the activation of certain genes involved in the increased generation of apoptosis. The pyridine-based chitosan thiosemicarbazones and their copper (II) complexes have shown a favorable structure for inhibiting the growth of tumorigenic MDCK and MCF-7 cancer cell lines (Adhikari et al., 2022). A more recent *in vitro* study showed that the combined use of isatin and 5-chloroisatin-based chitosan thiosemicarbazones displayed anticancer activity against MDCK and MCF-7 cancer cell lines (Adhikari et al., 2023). The sustained induction of apoptosis may lead to a significant reduction in the population of cancer cells, which can be achieved by the use of chitosan nanoparticles (Adhikari et al., 2022). The overall outcome was a reduction in cell viability, accompanied by the inactivation of cell division, growth promoter genes, and other virulence genes. In a previous study conducted by Rajivgandhi et al. (2020) and Wang et al. (2021), it was shown that chitosan nanoparticles exhibit anticancer activities against a range of cancer cells. Furthermore, the inhibitory effects were found to be dependent on the concentration of the nanoparticles.

Some limitations in this work need additional investigation. Firstly, it is necessary to examine the *in vitro* releasing profile of Api from Api@ChB NPs. Furthermore, due to limited financial resources, we were unable to conduct tunnel or flow cytometry experiments with annexin V must be performed anticancer results to demonstrate that apoptosis is occurring. Additionally, we recommend *in vivo* experiments in order to validate the effectiveness of the formulations we have developed.

## 4 Conclusion

In conclusion, bee chitosan is a promising novel natural carbohydrate polymer source that can be used as a carrier for many drugs and alternative compounds. In order to increase the efficiency of the chitosan, the ionic gelation method was used to prepare bee chitosan nanoparticles and then used as a carrier for encapsulating apitoxin. The dynamic light scattering properties; ζ-potentials and transmission electron microscopy results showed that the bee chitosan nanoparticles loaded with apitoxin exhibited good homogeneity and spherical size with white dots. The experimental data from the current study concluded that synthesized bee chitosan nanoparticles loaded with apitoxin exhibited greater antimicrobial activity against six common human pathogens, which were Gram-positive and Gram-negative bacterial and fungal strains. Although the nanoparticles were toxic at high concentrations, they had outstanding efficacy (~72% of cancer cells were eliminated) against the human colon cancer cell line (Caco2 ATCC ATP-37) and human liver cancer cell line (HepG2 ATCC HB-8065). This implied that they could be a viable choice for killing cancer cells at optimum levels.

## Data availability statement

The original contributions presented in the study are included in the article/Supplementary material, further inquiries can be directed to the corresponding authors.

## Ethics statement

Ethical approval was not required for the studies on humans in accordance with the local legislation and institutional requirements because only commercially available established cell lines were used. Ethical approval was not required for the studies on animals in accordance with the local legislation and institutional requirements because only commercially available established cell lines were used.

## Author contributions

MS: Conceptualization, Data curation, Methodology, Nano design, Validation, Visualization, Writing – original draft, Writing – review & editing. AZ: Data curation, Methodology, Validation, Visualization, Writing – original draft, Writing – review & editing. MA: Data curation, Funding, Methodology, Validation, Resources, Writing – review & editing. AM: Data curation, Funding, Methodology, Validation, Resources, Writing – review & editing. AS: Writing – review & editing. AbA: Writing – review & editing. AhA: Data curation, Funding, Methodology, Validation, Resources, Writing – review & editing. EA: Data curation, Funding, Methodology, Validation, Resources, Writing – review & editing. SZ: Data curation, Funding, Methodology, Validation, Resources, Writing – review & editing. NA: Data curation, Formal analysis, Funding acquisition, Investigation, Methodology, Validation, Visualization, Resources, Writing – review & editing. C-GL: Funding acquisition, Project administration, Supervisor, Writing – review & editing.
