# Supplementary material for: Bee chitosan nanoparticles loaded with apitoxin as a novel approach to eradication of common human bacterial, fungal pathogens and treating cancer
Source: Front Microbiol. 2024 Mar 15;15:1345478. doi: 10.3389/fmicb.2024.1345478 (PMC10978808; doi:10.3389/fmicb.2024.1345478)
Supplement: Supplementary file 1 [file Data_Sheet_1.docx]

**Appendix Supplementary data**


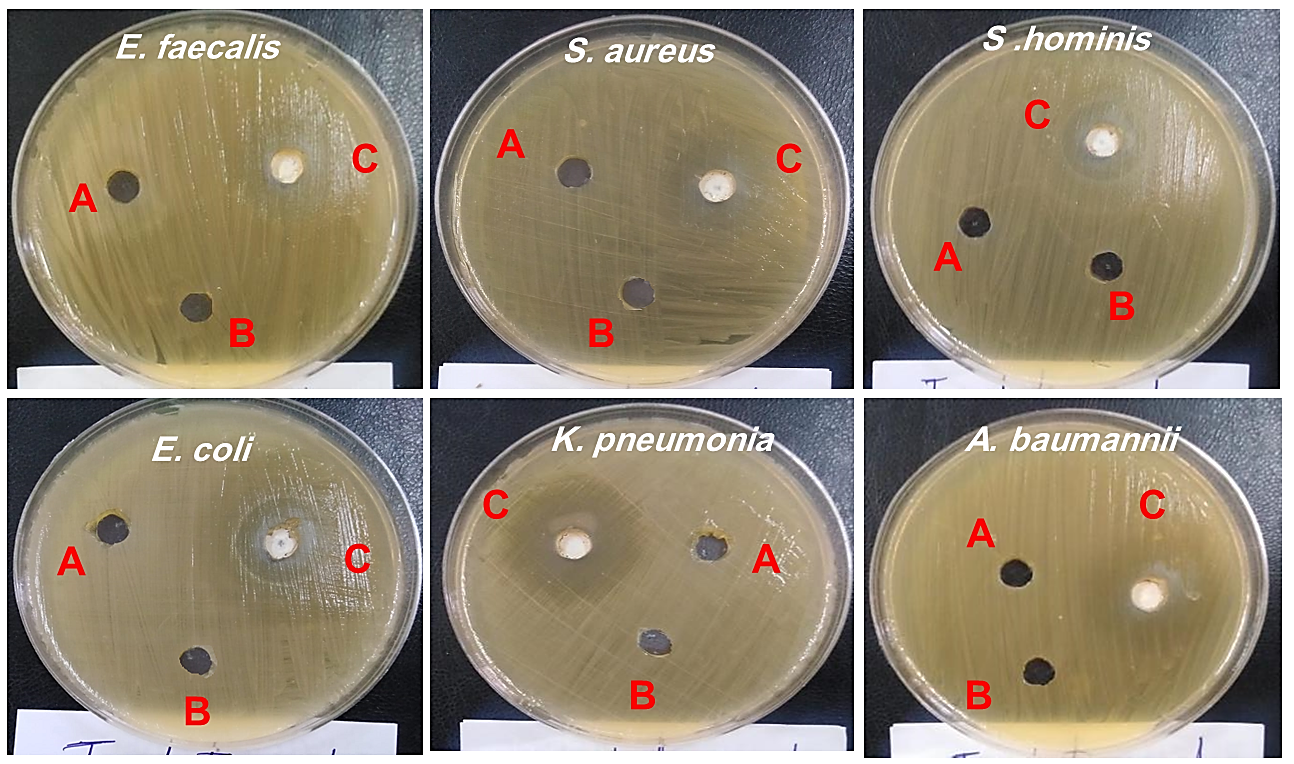


FigureS1. Positive and Negative control The inhibition zone produced against different pathogenic strains  *E. faecalis,*  *S. aureus*, *S .hominis, ,* *E. coli, K. pneumonia*, and *A. baumannii* and fungi against *C. auris*, and *A. niger* .(A)DMSO ;(B) ddH_2_O;and (c) gentamicin (MIC 8 µg mL-1)


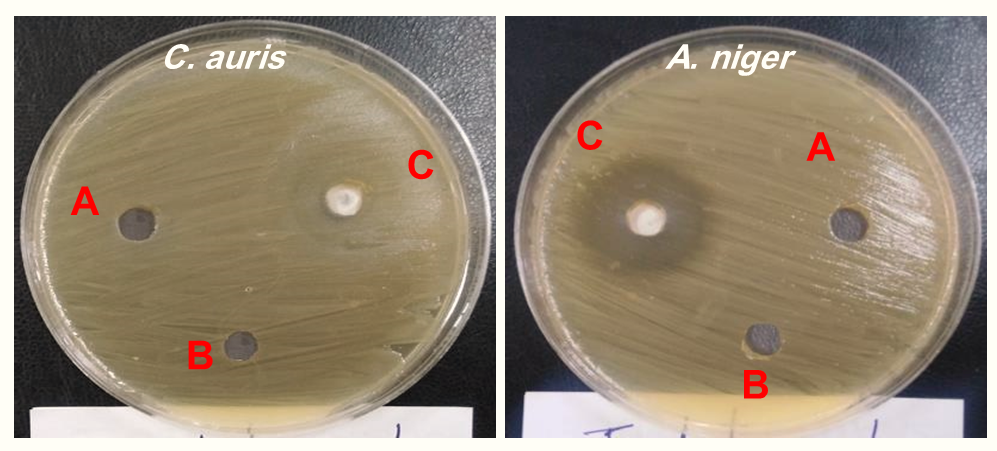


FigureS2. Positive and Negative control The inhibition zone produced against fungi *C. auris*, and *A. niger*.(A)DMSO ;(B) ddH_2_O;and (c) Fluconazole

**
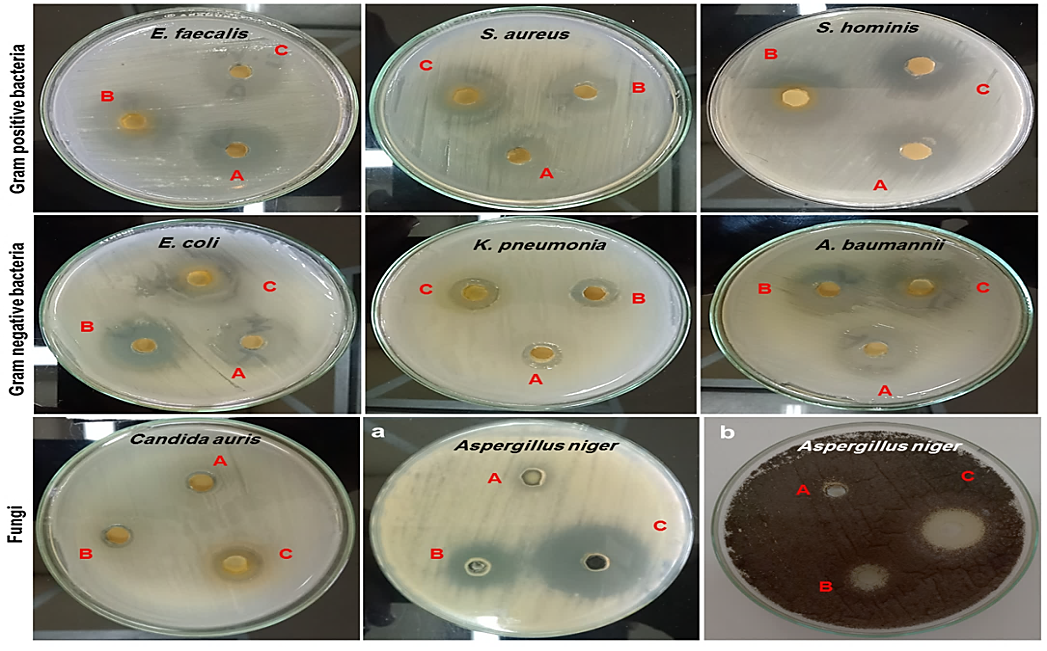
**

FigureS3. The inhibition zone produced against different pathogenic strains  *E. faecalis,*  *S. aureus*, *S .hominis,* *E. coli, K. pneumonia*, and *A. baumannii* ; antifungal activity against *C. auris*, (a) *A. niger* after 1 day of culture and (b) *A. niger* after 5 days of culture by various prepared samples (A) Api, (B) ChB NPs, and (C) Api@ChB NPs


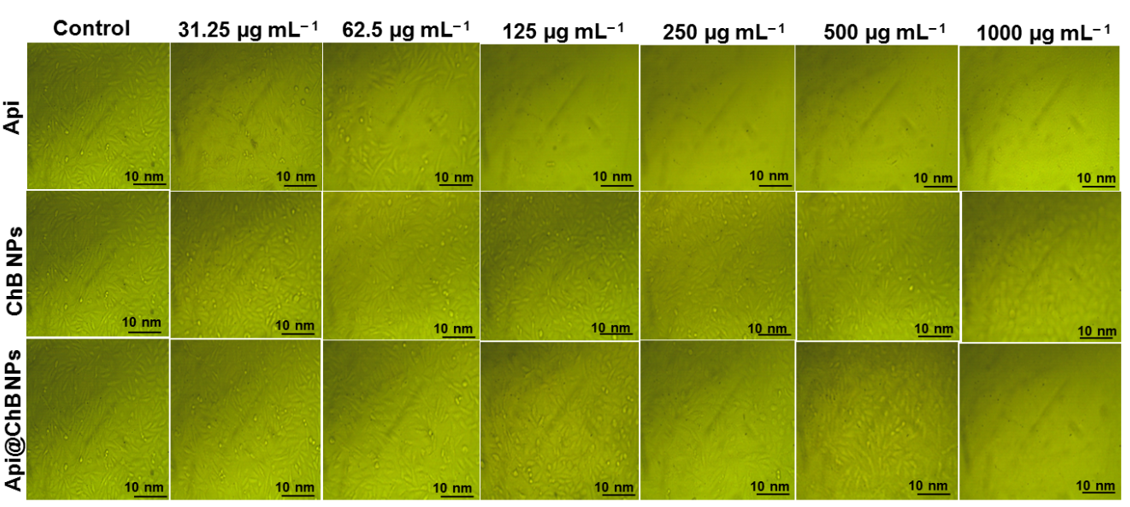


FigureS4. Morphological features for the effect of Api, ChB NPs and Api@ChB NPs on Vero ATCC CCL-81 cell lines. The images were taken from the cells were treated with an average size of 10 nm for 24 h.


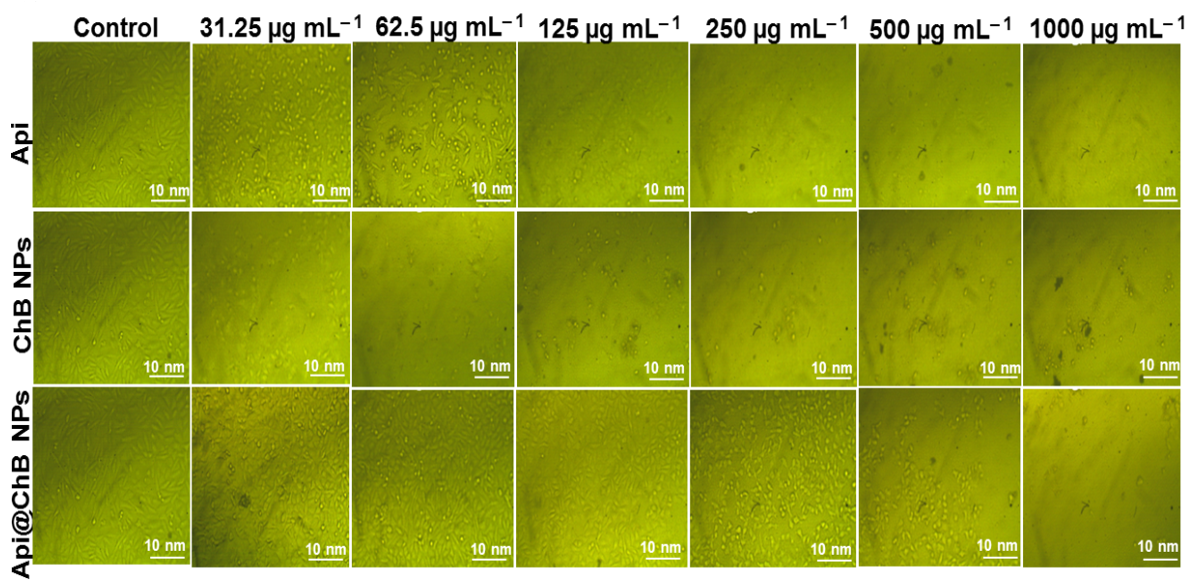


FigureS5. Morphological features for the effect of Api, ChB NPs and Api@ChB NPs on human colon cancer cells (Caco2 ATCC ATP-37). The images were taken from the cells were treated with an average size of 10 nm for 24 h.


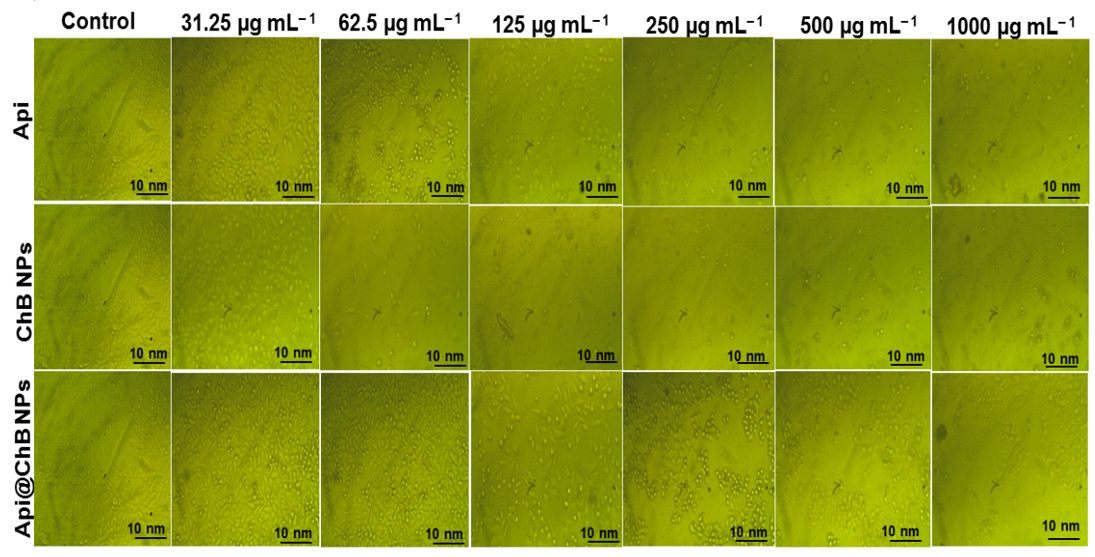


FigureS6. Morphological features for the effect of Api, ChB NPs and Api@ChB NPs on human Liver cancer cells (HepG2 ATCC HB-8065). The images were taken from the cells were treated with an average size of 10 nm for 24 h.
